# Regional Disparities in Rural Pension Provision: Examining Equity-Efficiency Dynamics in China’s Urbanization

**DOI:** 10.1093/geroni/igaf122.4184

**Published:** 2025-12-31

**Authors:** Huijing Kuang, Guoyong Ma, Hong Mi

**Affiliations:** Northeast Forestry University, Harbin, Heilongjiang, China (People’s Republic); Northeast Forestry University, Harbin, Heilongjiang, China (People’s Republic); Zhejiang University, Hangzhou, Zhejiang, China (People’s Republic)

## Abstract

Within the context of China’s delayed retirement policies, regional variations in demographic structures and social security provision have become increasingly pronounced. The regional equity-efficiency of the basic endowment insurance for urban and rural residents have emerged as pivotal issues in social security system reform. This study employs Suzhou (a high-tech industrial hub) and Daqing (a resource-dependent city) as comparative case studies. Utilizing demographic and social security data alongside policy document analysis, it investigates urban heterogeneity in social security implementation and reveals systemic issues concerning regional equity and efficiency. Key findings reveal: (1)Enrollment Scale Disparity: Daqing’s enrollment exceeds Suzhou’s by over 15-fold. Beyond rural residents, enrollees primarily comprise land-expropriated farmers generated during urban expansion and flexible workers transitioning from basic endowment insurance for employees;(2)Benefit Equity Gap: Substantial inter-city disparity exists in pension benefits. By 2025, Suzhou’s basic pension standard reached RMB 705/month, starkly contrasting with Daqing’s minimum standard of RMB 163/month—a gap exceeding fourfold;(3)Subsidy Efficiency Variation: Suzhou’s municipal subsidy for the highest contribution tier is 15%, fostering a virtuous “more contributions, higher returns” cycle, while constrained by local fiscal capacity, Daqing’s highest-tier subsidy ratio is only 3.5%, severely diminishing enrollment incentives;(4)Pension replacement rates exhibit polarization: For minimum-tier contributions, Suzhou’s rate represents 23.4% of rural residents’ per capita disposable income, compared to merely 9.8% in Daqing. The study proposes a scheme of “implementing the integration of basic pensions and adopting graded contribution subsidies” to narrow regional pension disparities and addressing issues of systemic equity and efficiency.

